# Production, Characterization, and Bioactivity of Fish Protein Hydrolysates from Aquaculture Turbot (*Scophthalmus maximus*) Wastes

**DOI:** 10.3390/biom10020310

**Published:** 2020-02-15

**Authors:** José Antonio Vázquez, Isabel Rodríguez-Amado, Carmen G. Sotelo, Noelia Sanz, Ricardo I. Pérez-Martín, Jesus Valcárcel

**Affiliations:** 1 Grupo de Biotecnología y Bioprocesos Marinos, Instituto de Investigaciones Marinas (IIM-CSIC), C/Eduardo Cabello, 6, CP 36208 Vigo, Galicia, Spain; carmen@iim.csic.es (C.G.S.); nsanz@iim.csic.es (N.S.); ricardo@iim.csic.es (R.I.P.-M.); jvalcarcel@iim.csic.es (J.V.); 2 Laboratorio de Reciclado y Valorización de Materiales Residuales (REVAL), Instituto de Investigaciones Marinas (IIM-CSIC), C/Eduardo Cabello, 6, 36208 Vigo, Galicia, Spain; 3 Department of Life Sciences of the International Iberian Nanotechnology Laboratory (INL), Avenida Mestre José Veiga, 4715-330 Braga, Portugal; isabel.rodriguez@inl.int; 4 Laboratorio de Bioquímica de Alimentos, Instituto de Investigaciones Marinas (IIM-CSIC), C/Eduardo Cabello, 6, 36208 Vigo, Galicia, Spain

**Keywords:** aquaculture by-products, turbot waste, valorization, fish protein hydrolysates, bioactive, circular bioeconomy

## Abstract

The valorization of wastes generated in the processing of farmed fish is currently an issue of extreme relevance for the industry, aiming to accomplish the objectives of circular bioeconomy. In the present report, turbot (*Scophthalmus maximus*) by-products were subjected to Alcalase hydrolysis under the optimal conditions initially defined by response surface methodology. All the fish protein hydrolysates (FPHs) showed a high yield of digestion (>83%), very remarkable degrees of hydrolysis (30–37%), high content of soluble protein (>62 g/L), an excellent profile of amino acids, and almost total in vitro digestibility (higher than 92%). Antioxidant and antihypertensive activities were analyzed in all cases, viscera hydrolysates being the most active. The range of average molecular weights (Mw) of turbot hydrolysates varied from 1200 to 1669 Da, and peptide size distribution showed that the hydrolysate of viscera had the highest content of peptides above 1000 Da and below 200 Da.

## 1. Introduction

One of the most critical challenges that humanity currently faces is the production of enough food for an expected population of 9.6 billion people by 2050. Regarding aquatic food, the world fish production reached 170 million tons in 2016, with around 48% obtained from aquaculture [1]. As traditional fishing stocks approach their maximum level of exploitation (in many cases, even overexploited), aquaculture arises as a natural evolution of fisheries for fish supply, showing great potential since the resources required to produce a kilogram of food suitable for consumption are less in the water than on the land [2]. Among the different cultivated species, turbot (*Scophthalmus maximus*) farming is remarkable from the economic, hedonistic, and health perspectives due to turbot market price and organoleptic and nutritive properties. China is the largest producer with an estimated 60,000 t per year, followed by Europe with 11,000 t in 2017 [1]. European production is concentrated in Galicia (northwest of Spain) and north of Portugal, monopolizing 75% and 21% of the continental output, respectively.

Although turbot is conventionally marketed as a whole fish, fillet presentations are growing year after year in Europe, recently achieving around 10%–15% of the total turbot sold. As a result, by-products generated in the filleting process (heads, trimmings, frames, and viscera) represent a new source of waste in aquaculture plants. This residual biomass accounts for about 60% of the total turbot weight and must be managed to avoid environmental health problems, ideally through valorization processes within the scope of circular bioeconomy principles [3]. The only previous study dealing with the valorization of turbot waste by Fang et al. [4] proposed turbot skin as a substrate for the fermentation of microorganisms, such as *Aspergillus oryzae*, to produce extracts rich in antioxidants. However, one the most valuable processes to achieve high protein recovery, including bioactive peptides, is by application of proteases under controlled conditions of operation, to digest fish waste and generate fish protein hydrolysates (FPHs) [5].

The enzymatic proteolysis of fish by-products using exogenous proteases is a fast, reproducible, and controllable way to separate the material into bones, oil, and an aqueous phase. This phase habitually presents a high content of different soluble proteins and peptides (of different sizes) as well as free amino acids [5,6]. Additionally, FPHs have demonstrated in many cases remarkable in vitro biological activities (antioxidant, antiproliferative, antihypertensive, etc.) and technological properties (emulsifying activity, foaming capacity, etc.) [7,8,9]. All these benefits make fish hydrolysates a valuable and easily digestible protein supplement, with high nutritional properties and interesting applications as an ingredient for human and animal functional foods [5,10,11]. From the 1940s, the number of fish species, types of fishery wastes, and proteolytic enzymes (both endo- and exogenous) studied for FPHs production has been extensive [11,12,13]. However, the application of this process to turbot waste remains unexplored.

The aim of this study was, initially, to optimize enzymatic proteolysis conditions to produce protein hydrolysates from a turbot farming processing by-product (heads). Subsequently, and based on the obtained optimal conditions, FPHs from other turbot wastes (viscera, trimmings + frames) were extensively produced at 5L-pH-stat scale evaluating hydrolysis kinetics and performing an exhaustive chemical characterization of the hydrolysates, including molecular weight and peptide size distribution along with antioxidant and antihypertensive properties. To the best of our knowledge, this is the first article studying the valorization of turbot waste by the production of enzymatic hydrolysates and associated functional responses.

## 2. Materials and Methods

### 2.1. Turbot by-Products

By-products of turbot (*Scophthalmus maximus*) generated from fish filleting were kindly supplied by Prodemar (Stolt Sea Farm S.A., Carnota, A Coruña, Spain). These materials (20–25 kg of each by-product) were frozen and kept at −18 °C until processing (Figure S1, Supplementary Materials). The three types of substrates were heads of turbot (Tu_H), trimmings and frames of turbot (Tu_TF), and viscera of turbot (Tu_V). Initially, they were ground in a meat mincer (Figure S1, Supplementary Materials) before hydrolysis.

### 2.2. Optimization of Protease Hydrolysis of Turbot by-Products

In order to know the best conditions of enzyme digestion, rotatable second order designs (with 5 replicates in the center of the experimental domain) were conducted studying the impact of temperature (T) and pH on the Alcalase 2.4L (2.4 AnsonUnit/g, AU/g enzyme, Nordisk, Bagsvaerd, Denmark) hydrolysis of Tu_H. Natural and codified values for factorial experiments jointly with the constant conditions applied for the agitation, enzyme concentration, and solid:liquid ratio (S:L) are shown in Table S1 (Supplementary Materials). The responses (dependent variables, Y) were the concentration of soluble protein (Prs), the maximum hydrolysis (*H_m_*), and the yield of digestion (V_dig_). Orthogonal least-squares calculation on factorial design data was used to obtain empirical equations describing the different responses assessed (Y) in function of the independent variables:

(1) $$Y=b_{0}+\sum_{i=1}^{n}b_{i}X_{i}+\sum_{\underset{j>i}{i=1}}^{n-1}\sum_{j=2}^{n}b_{ij}X_{i}X_{j}+\sum_{i=1}^{n}b_{ii}X_{i}^{2}$$

where Y is the dependent variable evaluated, b_0_ is the constant coefficient, b_i_ is the coefficient of linear effect, b_ij_ is the coefficient of combined effect, b_ii_ is the coefficient of quadratic effect, n is the number of variables, and X_i_ and X_j_ are the independent variables studied in each case.

Student’s t-test (α = 0.05) was employed to determine the statistical significance of coefficients. Coefficient of determination (R^2^) and adjusted coefficients of determination ($R_{adj}^{2}$) were used to establish goodness-of-fit, and the following mean squares ratios from Fisher F test (α = 0.05) were calculated to define model consistency: F1 = Model/Total error, being the model acceptable when $F1\geq F_{den}^{num}$; F2 = (Model + Lack of fitting)/Model, being the model acceptable when $F2\leq F_{den}^{num}$; and F3 = Total error/Experimental error, being the model acceptable when $F3\leq F_{den}^{num}$. $F_{den}^{num}$ are the theoretical values to α = 0.05 with corresponding degrees of freedom for numerator (num) and denominator (den). These experiments were carried out in a pH-Stat system equipped with a 100 mL enzyme reactor including temperature and agitation control.

Based on the optimal values of T and pH obtained in the above factorial plan, individual effects of enzyme concentration and (S:L) ratio on turbot heads hydrolysis were separately studied using the same equipment. In all optimization experiments, after hydrolysis (3 h) the content from mini reactors were centrifuged (15,000× *g*/20 min), the sediments (mainly bones) and supernatants were quantified, and FPHs were quickly heated (90 °C/15 min) for enzyme inactivation.

### 2.3. Production of Fish Protein Hydrolysates (FPHs) of Turbot by-Products

**Figure 1:**
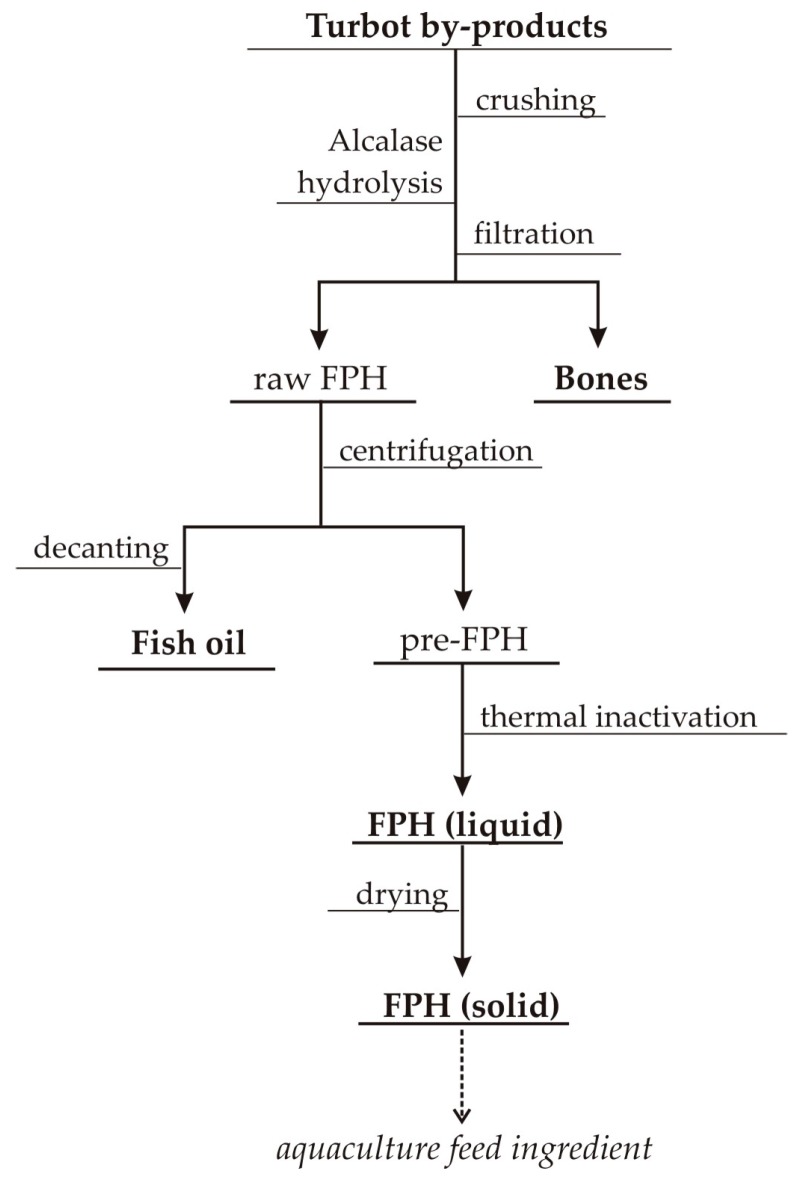
Schematic flowchart of turbot by-products processed through enzymatic hydrolysis.

Hydrolysis of turbot wastes was scaled-up and performed (ten independent batches for each substrate) in a controlled pH-Stat system with a 5 L glass-reactor using the optimal conditions defined for Tu_H. The alkaline reagent for pH-control was 5 M NaOH. Two kilograms of milled substrates were mixed with 2 L of distilled water and 0.2% (*v*/*w*) of Alcalase, and hydrolyzed for 3 h at 60 °C and pH 8.5. At the end of the hydrolysis, the content of the reactors was filtered (100 μm) to remove bones, the liquid hydrolysates were centrifuged (15,000× *g*/20 min) to recover oils (adding a step of decantation for 15 min), and protease deactivation was achieved by heating (90 °C/15 min) of FPHs (Figure 1). Solid FPH were obtained by freeze-drying. The time-course of hydrolysis was recorded by means of the degree of hydrolysis (H, as %) using the pH-Stat method [14] and modeled by the following Weibull equation [13]:

(2) $$H=H_{m}\left\{1-\exp\left[-\ln2{\left(\frac{t}{\tau }\right)}^{\beta }\right]\right\}\;\mathrm{with}\;v_{m}=\frac{\beta H_{m}\ln2}{2\tau }$$

where H is the degree of hydrolysis (%), t is the time of hydrolysis (min), H_m_ is the maximum degree of hydrolysis (%), β is a parameter related with the maximum slope of muscle hydrolysis (dimensionless), v_m_ is the maximum rate of hydrolysis (% min^−1^), and τ is the time required to achieve the semi-maximum degree of hydrolysis (min). The yields of digestion/liquefaction (V_dig_) of raw material to the liquid phase were also determined [13].

### 2.4. Chemical and Biological Determinations

The chemical composition of by-products was determined as (1) water, ash, and organic matter content [15]; (2) total nitrogen [15] and total protein as total nitrogen ×6.25; and (3) total lipids [16]. The profile of fatty acids from turbot oil was analyzed by gas chromatography after chemical methylation [17]. The basic analyses of FPHs were: (1) total soluble protein [18]; (2) total sugars [19]; (3) total protein as total nitrogen × 6.25 [15]; (4) amino acids content following the method of Moore et al. [20], employing an Amino Acid Analyser (Biochrom 30 series, Biochrom Ltd., Cambridge, UK); and (5) in vitro digestibility (pepsin method: AOAC Official Method 971.09) according to the modifications reported by Miller et al. [21].

Molecular weights of FPHs (>1 kDa) were determined by gel permeation chromatography (GPC). The system used was an Agilent 1260 HPLC consisting of quaternary pump (G1311B), injector (G1329B), column oven (G1316A), refractive index (G1362A), diode array (G1315C), and dual-angle static light scattering (G7800A) detectors. Standard and samples were eluted with a 0.15 M ammonium acetate/0.2 M acetic acid buffer at pH 4.5 pumped at 1 mL/min through four columns (PSS, Germany): Proteema precolumn (5 μm, 8 × 50 mm), Proteema 30 Å (5 μm, 8 × 300 mm), Proteema 100 Å (5 μm, 8 × 300 mm), and Proteema 1000 Å (5 μm, 8 × 300 mm) after a 100 μL injection. Column oven and light scattering detector were kept at 30 °C, and refractive index detector was maintained at 40 °C. Detectors were calibrated with a polyethylene oxide standard (PSS, Germany) of 106 kDa (Mw) and polydispersity index 1.05. Absolute molecular weights were estimated with refractive index increments (dn/dc) of 0.185. In the case of molecular weight of peptides from FPHs (<10 kDa), the samples of FPHs, after processing by centrifugation on Amicon-1 kDa (MerckMillipore, Darmstadt, Germany), were quantified by HPLC (220 nm UV-detection) using Superdex peptide 10/300 GL column (GE Healthcare Life Sciences, Little Chalfont, UK) with 0.1% trifluoroacetic acid in 30% of acetonitrile as mobile phase (flow rate of 0.4 mL/min) at 25 °C. The standards used were Blue Dextran (2 MDa), Cytochrome c (12.4 kDa), Aprotinin (6.5 kDa), Angiotensin II (1046 Da), Leucine encephalin (555 Da), Val-Tyr-Val (379 Da), and Gly-Gln (221 Da).

Biological activities as antihypertensive and antioxidant (AO) values were quantified in FPHs samples as (a) in vitro Angiotensin I-converting enzyme (ACE) inhibitory activity (IACE) according to the protocol of Amado et al. [22] calculating IC_50_ values (protein-hydrolysate concentration that generates a 50% IACE) by dose-response modeling; (b) 1,1-diphenyl-2-picrylhydrazyl (DPPH) radical-scavenging ability following a microplate protocol [23]; (c) ABTS (2,2′-azinobis-(3-ethyl-benzothiazoline-6-sulphonic acid) bleaching method at microplate scale [23]; (d) crocin bleaching assay also employing an optimized microplate protocol [24]. All antihypertensive and AO determinations were done in triplicate using FPHs samples at a concentration of 1 g/L of soluble protein.

### 2.5. Numerical and Statistical Analyses

Data fitting procedures and parametric estimations were conducted by minimization of the sum of quadratic differences between observed and model-predicted values, using the non-linear least-squares (quasi-Newton) method provided by the macro ‘Solver’ of the Microsoft Excel spreadsheet. Confidence intervals from the parametric estimates (Student’s t-test) and consistency of mathematical models (Fisher’s F test) were evaluated by “SolverAid” macro. The significance of comparisons between samples was analyzed by ANOVA with a significance level of *p* < 0.05.

## 3. Results and Discussion

The chemical composition of turbot by-products is listed in Table S2. The moisture of these materials varied from 64% to 73%, reaching 27% of organic matter in Tu_V and 9% of ash in Tu_TF. Higher contents of total lipids and total proteins were found in viscera and head, respectively. After defatting, viscera showed the greater level of proteins.

### 3.1. Optimization of Enzyme Hydrolysis of Turbot by-Products

**Figure 2:**
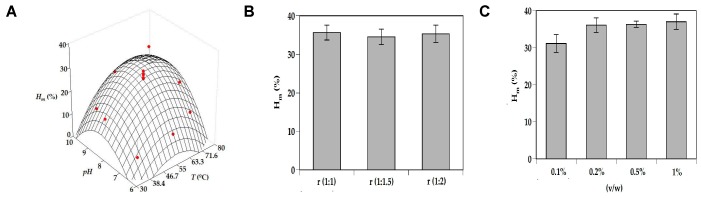

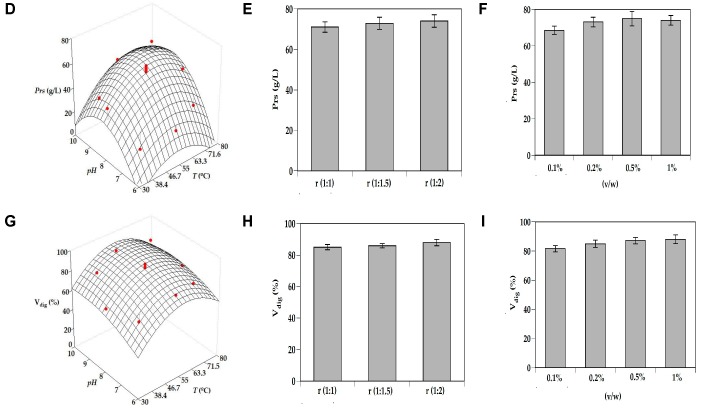
Optimization studies of heads-of-turbot (Tu_H) hydrolysis by Alcalase. Experimental data and predicted response surfaces describing the joint effect of pH and *T* on *H_m_* response (**A**), Prs response (**D**), and V_dig_ response (**G**) as defined in Table 1. (**B**) Individual effect of S:L ratio on *H_m_*; (**C**) individual effect of Alcalase concentration on *H_m_*; (**E**) individual effect of S:L ratio on Prs; (**F**) individual effect of Alcalase concentration on Prs; (**H**) individual effect of S:L ratio on V_dig_; (**I**) individual effect of Alcalase concentration on V_dig_. Error bars are the intervals of confidence for *n* = 2 (replicates of different hydrolysates) and α = 0.05.

In the first step, the experimental conditions of Alcalase hydrolysis on turbot heads were optimized by response surface methodology according to a two-variable factorial design for pH and temperature (Table S1, Supplementary Materials) combined with subsequent one-variable optimizations for the other independent variables: enzyme concentration and (S:L) ratio. The graphical description of the results for both approaches is displayed in Figure 2. Response surfaces including experimental data of Prs, V_dig_, and *H_m_* together with values predicted by polynomial equations (Table 1) are shown in A, D, and G plots. The agreements between simulated and experimental data were remarkable, with values of $R_{adj}^{2}$> 0.832. Fisher test (F1, F2 and F3) results were acceptable in all cases (data not shown). From values of Table 1, the average data of *pH_opt_* and *T_opt_* were calculated as 8.82 and 60.3 °C, respectively.

Taking into account these optimal values of pH and T, the effects of S:L ratio on numerical responses were individually studied (Figure 2B,E,H plots). The results were similar at all ratios tested without significant variations among them (*p* > 0.05). In order to reduce the use of water, a 1:1 ratio was hence chosen. Next, enzyme concentration effect was also evaluated (Figure 2C,F,I plots), obtaining significant best responses of Prs, V_dig_, and *H_m_* for concentrations higher than 0.1% *v*/*w* (*p* < 0.05). Based on all these outcomes, the optimal conditions found for Tu_H were finally: Alcalase 0.2% (*v*/*w*), ratio of (1:1), pH = 8.82, T = 60.3 °C, 200 rpm, 3 h of hydrolysis. These experimental conditions were applied to the rest of turbot by-products and the scaling of the FPHs.

In the last decade, Alcalase has shown an excellent capacity to hydrolyze several fish wastes such as salmon by-products [25], yellowfin tuna heads [26], and Atlantic cod, having being also applied to cattle viscera [12,27]. Other marine materials, e.g., cartilage and squid pens, have also been successfully digested by Alcalase [28,29], reducing operational costs and increasing effectiveness in the production of the biopolymers chondroitin sulfate and chitosan. The production of valuable FPHs from whole and by-products of fish discards using Alcalase has also been demonstrated [13,30]. Using a similar experimental approach, the optimized conditions for the hydrolysis of blue whiting were stablized as Alcalase 1% (*v*/*w*), ratio of (1:2), pH = 8.65, 60 °C, 200 rpm, 4 h of hydrolysis. Recently, enzymatic hydrolysates of salmonid by-products were produced by a procedure similar to the turbot ones: Alcalase 0.1% (*v*/*w*), ratio of (1:1), pH = 8.27, 56.2 °C, 200 rpm, t_h_ =3 h and Alcalase 0.2% (*v*/*w*), ratio of (1:1), pH = 8.98, 64.2 °C, 200 rpm, t_h_ = 3 h for salmon and rainbow trout heads, respectively [31].

### 3.2. Production and Chemical Composition of Turbot FPHs

**Figure 3:**
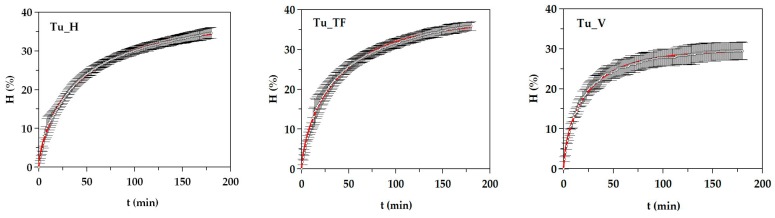
Alcalase hydrolysis of turbot by-products. Tu_H: heads of turbot, Tu_TF: trimmings and frames of turbot, and Tu_V: viscera of turbot. Experimental data of kinetics (symbols) were fitted to the Weibull equation (continuous line). Error bars are the confidence intervals for *n* = 10 (replicates of different hydrolysates) and α = 0.05.

Hydrolysates of turbot wastes were produced in 5L-pH-stat reactors under the conditions defined in the previous section. Kinetics of hydrolysis degree showed hyperbolic trends (Figure 3) perfectly modeled by equation (2). In this regard, the concordance among experimental and theoretical data was almost complete (*R*^2^ > 0.998) and the robustness of the equation was in all cases validated (*p* < 0.005) (Table S3, Supplementary Materials). The values of *H_m_* and τ were higher in Tu_H and Tu_TF hydrolysis, whereas the maximum rate was higher in Tu_V. No comparison can be made with literature results since this is the first report dealing with the production of FPHs from turbot from the aquaculture industry. Regarding other fish by-products, our outcomes of *H_m_* are in range (similar or slightly lower) with the values observed for hydrolysates from whole fish discards as megrim, blue whiting, red scorpionfish, and grenadier [30], but higher than those obtained for FPHs of boardfish, and pouting and by-products from salmon and trout [6,13,32,33].

Figure S2 (Supplementary Materials) shows different pictures related to the production of FPHs and the concomitant bones and oil recovered using the scheme reported in Figure 1. The amount of bone separated achieved 10% and 17% (*w*/*w* of the initial substrate) in Tu_TF and Tu_H, respectively, and no inorganic fraction was released from viscera (Table 2). The volume of oil extracted from turbot wastes was lower than expected, maybe due to the presence of oils as emulsions in FPH phases, which centrifugation was incapable of breaking. Oleic acid (around 34% *v*/*w* of the initial substrate) and palmitic acid (around 20% *v*/*w*) were the primary fatty acids present in oils. Still, the percentage of DHA and EPA was lower than 6% *v*/*w* (Table S4, Supplementary Materials). Regarding the omega-3/omega-6 ratio, values were slightly greater than 0.5, the figure set as threshold level to define the relevance of oils to be included in nutraceutical products [34].

The yields of digestion achieved by Alcalase on turbot wastes were very promising (95% on viscera in the best case) and similar to the outcomes obtained using fish discards as substrates [13,30]. In all cases, protein contents were higher than 61, 63, and 61 g/L when Prs, Pr-tN, and Σaa were determined in the final FPHs (Table 2 and Table 3), with significant differences between viscera and the two other materials (*p* < 0.05). The data of digestibilities were superb and identical for the three hydrolysates (higher than 92%). Glycine, glutamic acid, aspartic acid, and alanine were the predominant amino acids in turbot FPHs with remarkable content in the essential ones: TEAA/TAA values were higher than 28% (Table 3). Considering the concentrations of total and soluble proteins and the profile of amino acids along with the values of digestibility, valuable applications of turbot hydrolysates can be envisaged such as an ingredient in human food supplements and animal feed [10,35]. Further trials should be conducted to assess these potentialities. In fact, one of the main motivations to carry out the present production of turbot FPHs is for the subsequent application of these hydrolysates in the formulation of specific diets for the growth of culture fish as trout, salmon, and seabream.

**Figure 4:**
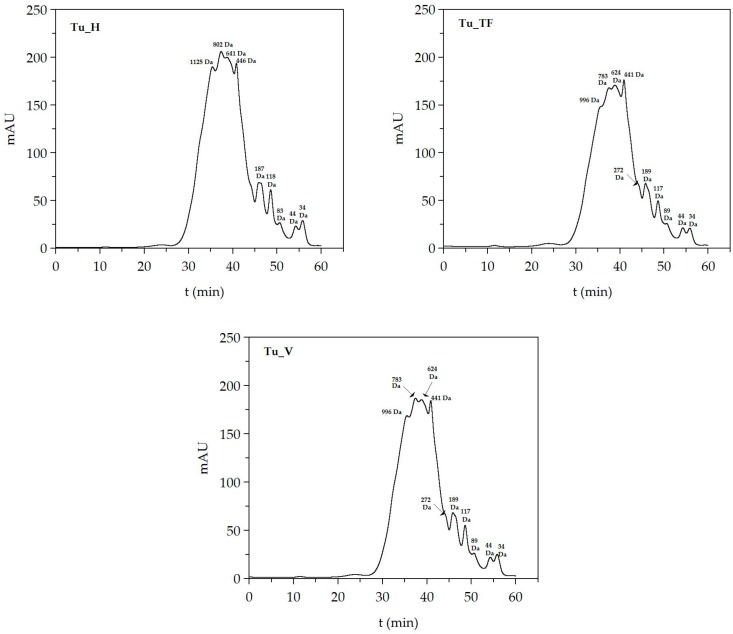
Size exclusion chromatographic profiles of turbot hydrolysates.

Regarding the size of the peptides present in the hydrolysates (Table 4), the average molecular weights (Mw) were 1622 ± 146 Da (PDI: 1.53), 1200 ± 53 Da (PDI: 1.45), and 2146 ± 144 Da (PDI: 2.44) for Tu_H, Tu_TF, and Tu_V, respectively. The most repeated peptide sizes for each FPHs (expressed as the number average molecular weight, Mn) were 1062 ± 102 Da for Tu_H, 826 ± 42 Da for Tu_TF, and 878 ± 64 Da for Tu_V. Combining the two chromatographic protocols described in the Materials and Methods Section, the distribution of peptide sizes are compiled in Table 4, and the graphical profiles of low peptides are also represented in Figure 4. In Tu_V, the content of peptides above 1 kDa was higher (65%) than those present in Tu_H and Tu_TF (54% and 45%, respectively). A concordance was observed between the molecular weight profiles and maximum degree of hydrolysis, supporting lower values of *H_m_* for bigger peptide size. Nevertheless, the largest fraction of peptides ranging 0–200 Da was found in hydrolysates of viscera.

### 3.3. Antioxidant and Antihypertensive Properties of Turbot Hydrolysates

The hydrolysis of turbot by-products yielded FPHs with bioactive properties. All studied FPH showed potential in vitro antioxidant and antihypertensive activities in the form of DPPH and ABTS scavenging abilities, Crocin bleaching effect, and ACE-inhibitory capacity (Table 5). The hydrolysates of viscera showed higher numerical antioxidant effect than Tu_TF and Tu_H, but in the case of ABTS and DPPH, the differences between samples were not significant (*p* > 0.05). Because this work is the first reference studying the production of FPHs from turbot materials, comparisons with other published reports cannot be made. However, the antioxidant capacity of turbot hydrolysates was not especially remarkable in comparison with hydrolysates of salmon, trout, or herring [36,37,38].

The ACE-inhibitory activity depended on the substrate hydrolyzed and the corresponding molecular weight profiles of FPHs. The inhibition data (as *I_ACE_*) varied from 53% to 82% with maximum response in Tu_V. In agreement with these values, FPHs from several by-products of fish discards (boardfish, red scorpionfish, and blue whiting) led to percentages of *I_ACE_* ranging 45%–70% [32,39,40]. Concerning the IC_50_ results, the strongest antihypertensive activity was observed in Tu_V (212.7 μg/mL) while in samples of head and trimmings these dose-response parameters were five-times higher. Hydrolysates from the muscle of hake (165 μg/mL), skins of gurnard (152 μg/mL), pouting (211 μg/mL), and red scorpionfish (226 μg/mL) revealed similar properties than our Tu_V [13,41]. In summary, FPHs from turbot viscera showed the highest bioactivity, both antioxidant and antihypertensive, which could probably be related to the presence of highest percentage of largest peptides (>1 kDa) (Table 4). Similar findings were also described for other fish enzyme hydrolysates: peptides obtained from substrates of cod, tuna, tilapia, Alaska pollock, and hoki and ranging from 1300 to 1800 Da revealed bioactive properties including those studied here [42,43,44,45,46].

## 4. Conclusions

In the present work, the valorization of by-products generated from aquaculture turbot filleting (heads, viscera, and trimmings + frames) has been reported for the first time. Thus, the hydrolysis of those substrates by Alcalase 2.4L on previously optimized conditions (0.2% (*v*/*w*), pH = 8.82, T = 60.3 °C, S:L = 1:1, t_h_ = 3 h and 200 rpm) led to the recovery of fish oils, clean bones, and the production of fish protein hydrolysates. The characterization of these FPHs indicated excellent chemical properties in terms of a high degree of hydrolysis, valuable concentration of soluble protein, adequate balance of amino acids for nutritional applications (relevant TEAA/TAA ratios), and almost complete in vitro digestibilities. Moreover, the in vitro antioxidant and antihypertensive ability of turbot hydrolysates, mainly from viscera, was remarkable. Bearing in mind these outcomes, FPHs from turbot could have useful applications as an ingredient in the formulation of human protein concentrates, pet food diets, and aquaculture feeds as a substitute of fish meals. Finally, the process developed here complies with the aims of the circular bioeconomy; however, these studies should be completed with further determinations of life cycle assessment (LCA) and CO_2_ footprint for the production of turbot FPHs.

## Figures and Tables

**Table 1:** Polynomial equations describing the combined effect of temperature (*T*) and *pH* on Alcalase hydrolysis of Tu_H. The coefficient of determination and adjusted determination (*R*^2^ and $R_{adj}^{2}$) and F-values are also shown. NS: non-significant. Optima values of the two variables (*T_opt_*, *pH_opt_*) to achieve the theoretical maximum responses (*Y_max_*) from the empirical equations were also calculated. S: significant; NS: not significant.

Parameters	*H_m_* (%)	*V_dig_* (%)	*Prs* (g/L)
***b_0_* (intercept)**	28.36 ± 1.57	88.59 ± 2.13	58.69 ± 2.27
***b_1_* (*T*)**	3.12 ± 1.25	3.70 ± 1.69	5.28 ± 1.80
***b_2_* (*pH*)**	4.74 ± 1.24	5.05 ± 1.69	10.0 ± 1.80
***b_12_* (*T*** **x** ***pH*** **)**	2.07 ± 1.76	NS	3.25 ± 2.54
***b_11_* (*T*^2^)**	−5.81 ± 1.34	−10.26 ± 1.82	−9.19 ± 1.94
***b_22_* (*pH*^2^)**	−6.27 ± 1.34	−3.05 ± 1.82	−11.8 ± 1.94
$R^{2}$	0.879	0.888	0.904
$R_{adj}^{2}$	0.803	0.832	0.835
***F1***	10.19 $[F_{7}^{5}=3.97]\Rightarrow S$	15.85 $[F_{7}^{5}=3.97]\Rightarrow S$	13.13 $[F_{7}^{5}=3.97]\Rightarrow S$
***F2***	0.705 $[F_{5}^{8}=4.82]\Rightarrow S$	0.558 $[F_{5}^{8}=4.82]\Rightarrow S$	0.688 $[F_{5}^{8}=4.82]\Rightarrow S$
***F3***	6.012 $[F_{5}^{8}=4.82]\Rightarrow S$	5.669 $[F_{5}^{8}=4.82]\Rightarrow S$	5.998 $[F_{5}^{8}=4.82]\Rightarrow S$
***T_opt_* (°C)**	61.1	58.2	61.6
***pH_opt_***	8.62	9.17	8.68
***Y_max_***	29.9%	91.0%	62.1 g/L

**Table 2:** Mass balances and proximal analysis of the products obtained from Alcalase hydrolysates of turbot by-products.

FPHs	m_b_ (%)	V_oil_ (%)	V_dig_ (%)	Prs (g/L)	Pr-tN (g/L)	TS (g/L)	Dig (%)
**Tu_H**	16.8 ± 1.4 ^a^	0.25 ± 0.19 ^a^	86.9 ± 1.2 ^a^	73.5 ± 4.9 ^a^	73.9 ± 4.5 ^a^	1.26 ± 0.14 ^a^	92.1 ± 4.1 ^a^
**Tu_TF**	9.7 ± 1.4 ^b^	4.25 ± 1.09 ^b^	82.5 ± 1.2 ^b^	73.9 ± 3.8 ^a^	72.7 ± 3.9 ^a^	1.34 ± 0.17 ^a^	93.8 ± 2.4 ^a^
**Tu_V**	-	0.46 ± 0.10 ^a^	95.3 ± 1.0 ^c^	61.6 ± 2.8 ^b^	63.6 ± 2.9 ^b^	1.39 ± 0.25 ^a^	92.4 ± 1.9 ^a^

Shown errors are the confidence intervals for *n* = 10 (replicates of different hydrolysates) and α = 0.05. m_b_: percentage of the bones recovered; V_oil_: percentage of oil recovered; V_dig_: yield of digestion process; Prs: total soluble protein determined by Lowry; Pr-tN: total protein determined as total nitrogen ×6.25; TS: total sugars; Dig: digestibility. Different letters in each column means significant difference between fish discards (*p* < 0.05).

**Table 3:** Amino acids content of fish protein hydrolysates (FPHs) (% or g/100 g total amino acids) from turbot by-products. OHPro: hydroxyproline. Pr: protein concentration calculated in g/L as the total sum of amino acids present in FPH. TEAA/TAA: ratio total essential amino acids for human/total amino acids. Errors are the confidence intervals for *n* = 10 (replicates of independent hydrolysates) and α = 0.05.

Amino Acids	Tu_H	Tu_TF	Tu_V
**Asp**	8.81 ± 0.16	9.63 ± 0.10	9.26 ± 0.42
**Thr**	3.64 ± 0.16	3.90 ± 0.06	3.85 ± 0.18
**Ser**	5.67 ± 0.14	5.37 ± 0.18	5.39 ± 0.36
**Glu**	12.86 ± 0.18	13.55 ± 0.08	13.12 ± 0.32
**Gly**	14.50 ± 0.32	12.57 ± 0.15	13.00 ± 0.50
**Ala**	8.38 ± 0.24	8.06 ± 0.05	7.83 ± 0.22
**Cys**	0.62 ± 0.05	0.77 ± 0.10	0.71 ± 0.11
**Val**	2.96 ± 0.09	3.21 ± 0.11	3.23 ± 0.12
**Met**	2.76 ± 0.15	2.90 ± 0.04	2.84 ± 0.17
**Ile**	1.97 ± 0.11	2.19 ± 0.08	2.29 ± 0.12
**Leu**	5.37 ± 0.11	5.93 ± 0.11	5.86 ± 0.17
**Tyr**	2.90 ± 0.16	3.18 ± 0.08	3.11 ± 0.20
**Phe**	4.16 ± 0.18	4.51 ± 0.17	4.31 ± 0.37
**His**	1.61 ± 0.07	1.75 ± 0.04	1.85 ± 0.11
**Lys**	5.57 ± 0.14	6.15 ± 0.11	6.18 ± 0.23
**Arg**	6.53 ± 0.24	6.34 ± 0.08	6.59 ± 0.36
**OHPro**	4.00 ± 0.19	3.14 ± 0.18	3.42 ± 0.35
**Pro**	7.67 ± 0.25	6.86 ± 0.13	7.14 ± 0.37
**Pr (Σaa) (g/L)**	68.62 ± 5.05	69.72 ± 4.39	61.94 ± 2.84
**TEAA/TAA (%)**	28.04	30.54	30.41

**Table 4:** Average molecular weights (as Mn and Mw) and associated confidence intervals for *n* = 5 (samples from different hydrolysates) and α = 0.05. Percentage of peptides distribution between molecular weight ranges was also determined. PDI: polydispersity index.

FPHs	Mn (Da)	Mw (Da)	PDI	0–0.2 kDa (%)	0.2–0.5 kDa (%)	0.5–1 kDa (%)	1–3 kDa (%)	>3 kDa (%)
**Tu_H**	1062 ± 102	1622 ± 146	1.527	8.1 ± 0.6	14.9 ± 0.0	23.1 ± 0.5	46.0	8.0 ± 1.4
**Tu_TF**	826 ± 42	1200 ± 53	1.453	7.0 ± 0.9	14.1 ± 0.2	34.1 ± 1.2	40.3	4.4 ± 0.9
**Tu_V**	878 ± 64	2146 ± 144	2.444	12.3 ± 3.9	6.6 ± 0.5	16.0 ± 1.2	52.9	12.21

**Table 5:** Bioactivities of FPHs from turbot by-products. Errors shown are the confidence intervals for *n* = 3–5 (samples from independent hydrolysates) and α = 0.05. Different letters in each column means significant difference between fish discards (*p* < 0.05).

Antioxidant				Antihypertensive	
FPHs	DPPH (%)	ABTS (μg BHT/mL)	Crocin (μg Trolox/mL)	I_ACE_ (%)	IC_50_ (μg protein/mL)
**Tu_H**	36.12 ± 2.81 ^a^	10.01 ± 0.98 ^a^	7.30 ± 0.92 ^a^	60.4 ± 5.8 ^a^	1273.6 ± 74.5 ^a^
**Tu_TF**	41.09 ± 0.98 ^b^	11.47 ± 2.14 ^a^	7.99 ± 0.51 ^a^	52.6 ± 24.1 ^a^	1063.4
**Tu_V**	65.15 ± 4.08 ^c^	12.81 ± 1.92 ^a^	8.03 ± 0.78 ^a^	81.9 ± 8.9 ^a,b^	212.7 ± 63.7 ^b^

## Supplementary Materials

The following are available online at https://www.mdpi.com/2218-273X/10/2/310/s1, Figure S1: Pictures of the turbot by-products (raw materials), Figure S2: Pictures describing the production of turbot hydrolysates, Table S1: Experimental design for the optimization of the FPHs, Table S2: Proximate composition of turbot by-products, Table S3: Kinetic parameters from Weibull equation modeling the time course of the hydrolysis degree, Table S4: Fatty acids content from fish oils recovered from different wastes of turbot.
