# Supplementary material for: Production, Characterization, and Bioactivity of Fish Protein Hydrolysates from Aquaculture Turbot (Scophthalmus maximus) Wastes
Source: Biomolecules. 2020 Feb 15;10(2):310. doi: 10.3390/biom10020310 (PMC7072122; doi:10.3390/biom10020310)
Supplement: Supplementary file 1 [file biomolecules-10-00310-s001.pdf]

Production, characterization and bioactivity of fish protein hydrolysates from aquaculture turbot (*Scophthalmus maximus*) wastes.

SUPPLEMENTARY MATERIAL

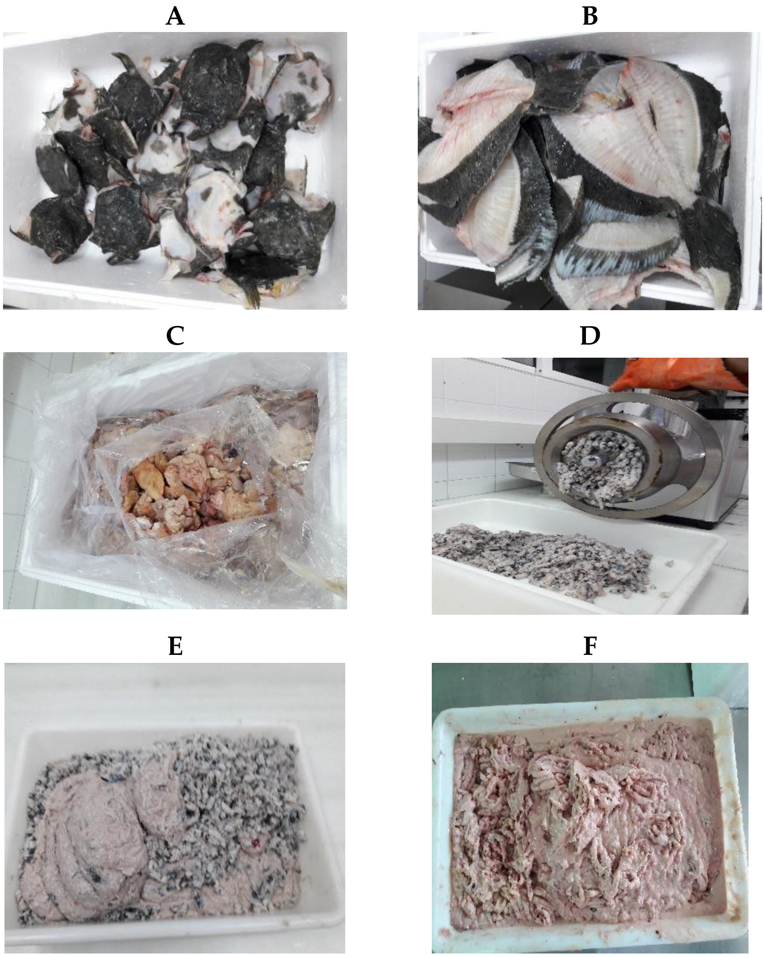

Figure S1. Pictures of initial and crushed turbot by-products: A) heads, B) trimmings and frames, C) viscera, D) minced heads. E) minced trimmings and frames and F) minced viscera.

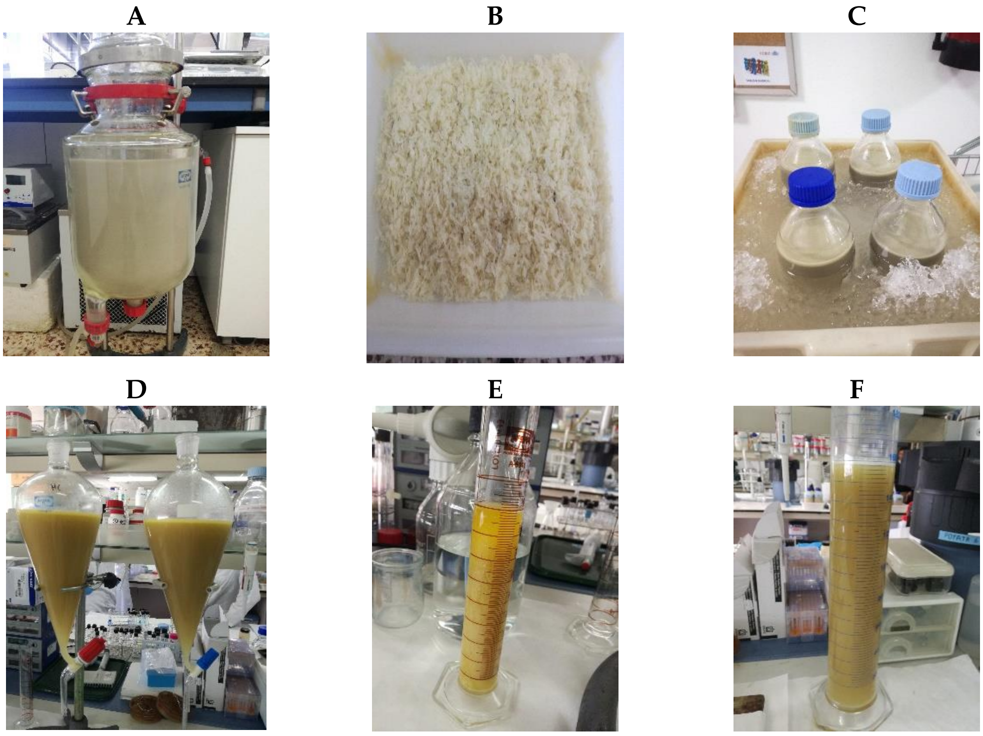

Figure S2. Different sequences of enzymatic hydrolysis of turbot wastes in a 5L-pH-stat reactor (A) with the differential recovery of clean bones (B) and fish oils (E) together with the production of liquid hydrolysate (F). The rest of the images show the cooling of hydrolysates prior centrifugation (C) and decantation of FPH to separate fish oil (D).

Table S1. Experimental domain and coding of the independent variables in the factorial design executed to study the joint effect of pH and temperature on the Alcalase hydrolysis of turbot heads (Tu_H).

	Natural values	
**Coded values**	**pH**	**T (ºC)**
-1.41	6.0	30.0
-1	6.6	37.3
0	8.0	55.0
+1	9.4	72.7
+1.41	10.0	80.0

Codification: $V_c = (V_n - V_0)/\Delta V_n$
Decodification: $V_n = V_0 + (\Delta V_n \times V_c)$
V_n = natural value of the variable to codify
ΔV_n = increment of V_n for unit of V_c
V_0 = natural value in the centre of the domain
V_c = codified value of the variable

Constant conditions
Agitation= 200 rpm
r (S:L)= 1:1
[Alcalase]= 0.5% (v/w) or 12 AU/kg of heads
time of hydrolysis= 3 h

Table S2. Proximate composition of turbot by-products in terms of moisture (Mo), organic matter (OM) and ashes (Ash). Total lipids (Lip), proteins (Pr-tN, as total nitrogen x 6.25) and proteins after degreasing samples (Pr-tN*) were determined using dried substrates. Error are the confidence intervals for n=3 (independent samples) and α=0.05. Different letters in each column means significant difference between fish discards ($p < 0.05$).

	Mo (%)	OM (%)	Ash (%)	Lip (%)	Pr-tN (%)	Pr-tN* (%)
**Tu_H**	72.7±0.6^{a}	20.6±0.3^{a}	6.8±0.9^{a}	5.9±0.2^{a}	65.2±1.6^{a}	73.3±1.5^{a}
**Tu_TF**	64.1±3.2^{b}	26.9±1.2^{b}	8.9±2.5^{a}	26.0±0.5^{b}	43.9±3.6^{b}	69.0±9.2^{a}
**Tu_V**	71.6±0.9^{a}	27.2±0.9^{b}	1.2±0.1^{b}	40.0±0.2^{c}	49.6±1.8^{c}	81.5±2.5^{b}

Table S3. Kinetic parameters and associated error (as intervals of confidence) obtained from Weibull equation modeling the time course of the hydrolysis degree (*H*) of turbot by-products catalysed by Alcalase. Determinaton coefficients (R^2) of fittings and p-values are also shown. Different letters in each column means significant difference between fish discards ($p < 0.05$).

	H_m (%)	α (dimensionless)	τ (min)	v_m (% min^{-1})	R^2	p-values
**Tu_H**	37.46±0.32^{a}	0.696±0.010^{a}	28.83±0.56^{a}	0.313±0.007^{a}	0.999	<0.005
**Tu_TF**	37.81±0.34^{a}	0.717±0.012^{a}	25.37±0.50^{b}	0.370±0.009^{b}	0.998	<0.005
**Tu_V**	29.78±0.12^{b}	0.699±0.011^{a}	13.68±0.19^{c}	0.528±0.008^{c}	0.998	<0.005

Table S4. Fatty acids (as %) presents in the fish oils recovered from turbot wastes complementary to the production of FPHs. Error are the intervals of confidence for n=3 and α=0.05.

Formula	Fatty acids	Tu_TF	Tu_H	Tu_V
**C14:0**	Myristic acid	5.88±0.55	5.52±0.30	5.71±0.35
**C15:0**	Pentadecanoic acid	0.56±0.15	0.64±0.12	0.64±0.10
**C16:0**	Palmitic acid	19.80±1.37	20.68±1.39	20.59±1.04
**C16:1n7c**	Palmitoleic acid	6.87±1.49	6.54±0.44	6.77±0.90
**C18:0**	Stearic acid	2.82±0.38	2.52±0.23	2.68±0.28
**C18:1n9c,t**	Oleic acid	34.14±5.60	33.54±1.68	33.22±2.92
**C18:2n6c,t**	Linoleic acid	12.89±2.25	12.15±0.97	12.51±1.20
**C18:3n3**	Linolenic acid	5.14±0.98	6.00±0.63	5.51±0.65
**C20:1n9**	Eicosenoic acid	3.15±2.16	2.56±0.38	3.25±1.15
**C20:2n6**	Eicosadienoic acid	1.57±0.30	1.53±0.23	1.61±0.21
**C20:4n6**	Arachidonic acid	1.36±0.44	1.32±0.15	1.38±0.18
**C21:4n3**	Heneicosatetraenoic acid	1.46±0.47	1.36±0.15	1.46±0.28
**C20:5n3**	Eicosapentaenoic acid (EPA)	0.46±0.17	0.45±0.06	0.48±0.10
**C22:6n3**	Docosahexaenoic acid (DHA)	3.87±2.27	5.19±0.47	4.19±1.38
	**DHA+EPA (%)**	4.34±2.12	5.65±0.43	4.67±1.30
	**r: ω-3 / ω-6**	0.71±0.18	0.87±0.11	0.77±0.14
